# An altered uterine microbiota with endometrial hyperplasia

**DOI:** 10.1186/s12866-024-03379-1

**Published:** 2024-07-12

**Authors:** Xue Ying, Gufeng Xu, Huiyun Wang, Yue Wang

**Affiliations:** Department of Ambulatory Surgery, Women’s Hospital, Zhejiang University School of Medicine, China. 1 Xueshi Road, Hangzhou, Zhejiang 310006 P.R. China

**Keywords:** Microbiome, Endometrial hyperplasia, Uterus, 16S rDNA, *Delftia*, *Serratia*, *Stenotrophomonas*

## Abstract

**Background:**

Endometrial hyperplasia (EH) is a precursor to endometrial cancer, and the role of the microbiome in its development is unclear.

**Results:**

The present study investigated the uterine microbiome in patients with benign uterine conditions and endometrial hyperplasia. A significant structural shift in the uterine microbiome of patients with endometrial hyperplasia compared to those with benign conditions was found. *Delftia*, *Serratia* and *Stenotrophomonas* were significantly enriched in endometrial hyperplasia samples and associated with the presence of endometrial hyperplasia.

**Conclusions:**

The novel finding suggested that increased abundance of *Delftia*, *Serratia* and *Stenotrophomonas* is associated with the presence of endometrial hyperplasia. Further investigation is needed to determine the value of these microbes as biomarkers for endometrial hyperplasia.

## Background

Endometrial hyperplasia (EH) is a precursor to endometrial carcinoma (EC), a common malignant tumor derived from the endometrium [1, 2]. In 2014, the World Health Organization (WHO) proposed a new classification of endometrial hyperplasia, dividing it into two categories: with or without atypical endometrial hyperplasia (AEH) or endometrial intraepithelial neoplasia (EIN) [3]. The etiology of EH and EC is still unclear. Recent evidence suggests that the uterine microbiota and specific bacterial species may be linked to the progression of endometrial hyperplasia and endometrial cancer [4]. Previously, the uterine cavity was believed to be sterile. However, with the development of next-generation sequencing of the 16S rRNA gene, it has become evident that the endometrium harbors a microbiota [5]. The endometrial microbiota consists of a variety of different microorganisms. When dysbiosis occurs, hallmarks of cancer, such as chronic inflammation, epithelial barrier breach, changes in cellular proliferation and apoptosis, genomic instability, angiogenesis, and metabolic dysregulation, can be impacted due to altered immunological and metabolic signaling [6].

Oncogenic viruses have been identified in various types of cancers, making them valuable biomarkers for tumor screening and prevention. One such virus is the Human papillomavirus (HPV), which is responsible for causing persistent infections that lead to cervical cancer [7]. HPV has been widely used as a biomarker for detecting cervical cancer, and HPV vaccines have been employed to prevent it [8]. Another example is Helicobacter pylori (H. pylori), a well-known carcinogen for gastric cancer [9]. Eradicating treatment for H. pylori has been widely utilized to reduce the incidence and mortality of gastric cancer [10]. Biomarkers such as oncogenic viruses or microbiota play an important role in cancer prevention and screening, and their identification and understanding may lead to the development of effective treatments and interventions. Previous study revealed the presence of A. vaginae and Porphyromonas sp. in the female reproductive tract, along with a high vaginal pH, has been found to be statistically associated with the presence of endometrial cancer [11]. However, currently, there is no widely accepted microbiota that has been found to be associated with the pathology of endometrial hyperplasia (EH) or endometrial cancer (EC).

Previous studies have investigated the diversity of microbiota in normal endometrium and EC/EH, revealing a notable contrast in microbiome composition. Interestingly, the diversity between benign endometrium and endometrial hyperplasia (EH) is even more significant. To further our understanding, our study aims to investigate the components of the microbiome in EH and identify microbiome-associated biomarkers that are related to this condition. Through this investigation, we hope to shed light on potential biomarkers for EH and contribute to advancements in the field of uterine microbiome research.

## Results

### Clinical information of the included patients

A total of 68 patients who underwent endometrial biopsy were included. Out of these patients, 53 women were diagnosed with benign endometrium while 15 women were diagnosed with endometrial hyperplasia. Among the 15 women diagnosed with hyperplasia, 13 had non-atypical hyperplasia while 2 had atypical hyperplasia. The final pathology was used to make all the diagnoses. The baseline characteristics of the two groups, such as age, BMI, abortion times, did not differ significantly, as indicated by all P-values being greater than 0.05, as shown in Table 1.

**Table 1:** Clinical characteristics of patients with normal endometrium or endometrial hyperplasia

	Normal endometrium (*N* = 53)	Endometrial Hyperplasia (*N* = 15)	*P* value
Age	44.32 ± 7.35	43.60 ± 4.58	0.72
BMI	25.04 ± 4.25	22.78 ± 2.36	0.06
Endometrial thickness	13.10 ± 5.00	11.16 ± 4.32	0.16
Menopause status			
Pre-menopause	46	15	0.14
Post-menopause	7	0	
Gravida			
0	2	0	0.45
> = 1	51	15	
Parity			
0	2	0	0.45
> = 1	51	15	
Hypertension			
Yes	9	3	0.79
No	44	12	
Diabetes			
Yes	2	1	0.63
No	51	14	
Smoking			
Never	52	15	0.59
Quit	1	0	
Current	0	0	
Vaginal PH	4.11 ± 1.33	4.75 ± 0.79	0.38
Indication for endometrial biopsy			
Abnormal vaginal bleeding^a^	46	15	0.14
Abnormal ultrasound finding^b^	7	0	
Hormone therapy within 10 years^c^			
Yes	4	1	0.90
No	49	14	
History of anovulation^d^			
Yes	14	7	0.15
No	39	8	

^a^Abnormal vaginal bleeding, such as postmenopausal bleeding or intermenstrual bleeding

^b^Asymptomatic with abnormal ultrasound finding, such as thick endometrium or heterogeneity of endometrium

^c^Hormone therapy such as OC (oral contraception)

^d^Anovulation is indicated by the menstrual cycle longer than 35 days

### Comparison of the composition of the microbiota between normal endometrium and endometrial hyperplasia

At the phylum level, patients with or without endometrial hyperplasia (EH) had a dominant presence of Proteobacteria, Firmicutes, and Bacteroidetes in their endometrial microbiome. Patients with EH showed an increased abundance of Proteobacteria, particularly Delfitia, compared to those with normal endometrium.

**Fig. 1:**
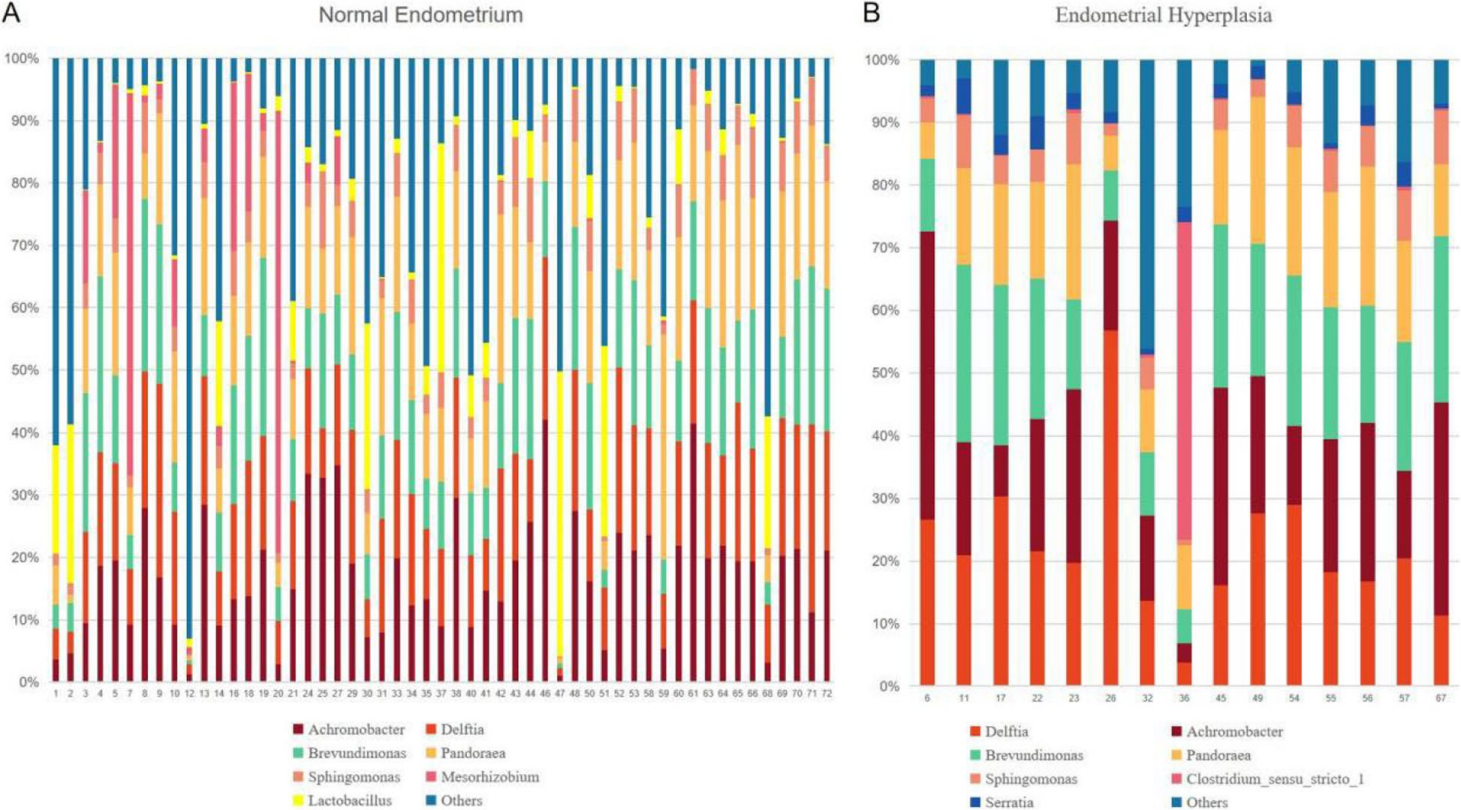
Bar charts of the bacterial species compositions in the endometrial microbiome. The top 7 bacterial species are displayed. One bar on the horizontal axis represents one sample. The vertical axis represents the bacterial abundance in the microbiota

At the genus level, the endometrial microbiome of patients with normal endometrium was mainly composed of *Achromobacter, Delftia, Brevundimonas, Pandoraea, Sphingomonas, Mesorhizobium*, and *Lactobacillus* (Fig. 1A). In contrast, patients with EH showed a dominance of *Delftia, Achromobacter, Brevundimonas, Pandoraea, Sphingomonas, Clostridium_sensu_stricto_1,* and *Serratia* (Fig. 1B).

*Achromobacter, Delftia, Brevundimonas, Pandoraea*, and *Sphingomonas* were the primary genera in both normal endometrium and EH. However, *Delftia* was more abundant in the endometrial microbiome of patients with EH. These findings suggest that alterations in the endometrial microbiome may play a role in the development of EH.

### Comparison of the diversity of the microbiota between normal endometrium and endometrial hyperplasia

**Fig. 2:**
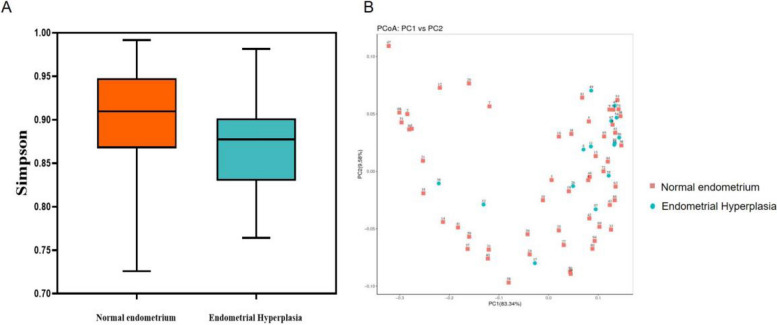
α and β diversity comparison of the endometrial microbiome between benign endometrium and EH. **A** α diversity comparison of the endometrial microbiome between benign endometrium and EH. Simpson index. **B** β-diversity comparison of the endometrial microbiome between benign endometrium and EH

The group with endometrial hyperplasia had significantly lower alpha diversity, as measured by the Simpson index (*P* = 0.023), when compared to the benign group (as shown in Fig. 2A). Furthermore, by calculating weighted UniFrac distance matrices and representing them as principal coordinates (as shown in Fig. 2B), we were able to identify significant differences in beta diversity between the two groups. These findings provide valuable insights into the potential role of microbiota in the development of endometrial hyperplasia, and highlight the importance of further investigation in this area.

### *Delftia*, *Serratia* and *Stenotrophomonas* were abundant in EH

**Fig. 3:**
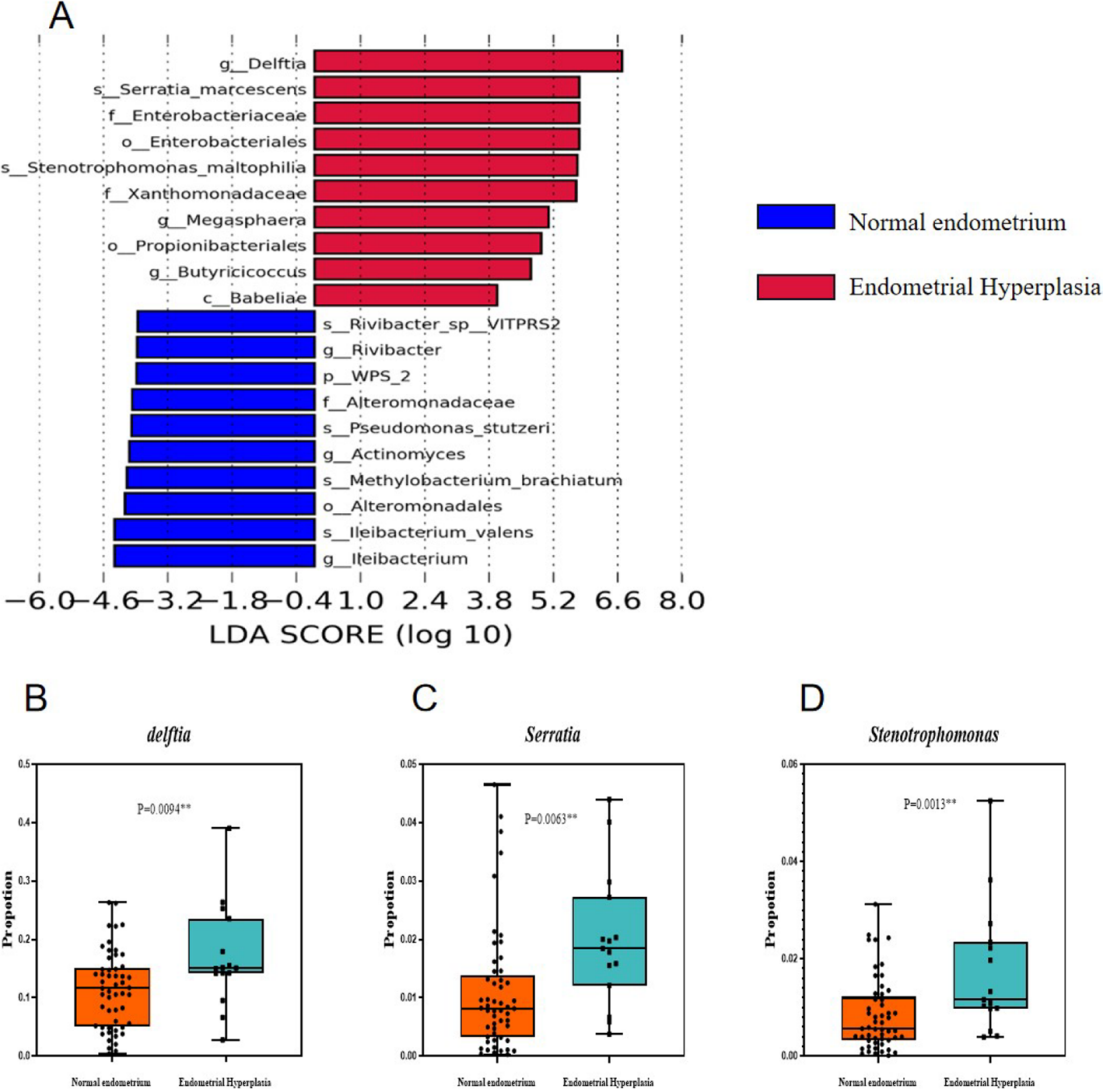
Significant differences in relative abundances between the normal endometrium and endometrial hyperplasia. **A** Histogram of LEfSe analysis of uterine flora between above groups. **B**, **C**, **D** Abundance of *Delftia*, *Serratia* and *Stenotrophomonas* between benign endometrium and EH

Significant differences in relative abundances between two groups were assessed at the genus level using the ANCOM-II method. The W statistic was used to determine significant differences in taxa relative to other taxa. The W statistics were then normalized with each total taxa number. Finally, our analysis revealed that *Delftia*, *Serratia* and *Stenotrophomonas* exhibited significant differences between the two groups, as shown in Fig. 3 and Table 2. The group with endometrial hyperplasia had higher abundances of *Delftia*, *Serratia* and *Stenotrophomonas* compared to the group with normal endometrium.

**Table 2:** Comparison of the abundance of the *Delftia, Serratia* and *Stenotrophomonas* in the endometrial microbiome of women in Group I (normal endometrium) and Group II (Endometrial Hyperplasia)

Index	Normal endometrium (*N* = 53)	Endometrial Hyperplasia (*N* = 15)	*P* value
*Delftia*	0.114 ± 0.066	0.170 ± 0.088	0.0094
*Serratia*	0.011 ± 0.011	0.020 ± 0.012	0.0063
*Stenotrophomonas*	0.008 ± 0.007	0.017 ± 0.013	0.0013

### Evaluation of the diagnostic accuracy of the *Delftia*, *Serratia* and Stenotrophomonas for EH

**Fig. 4:**
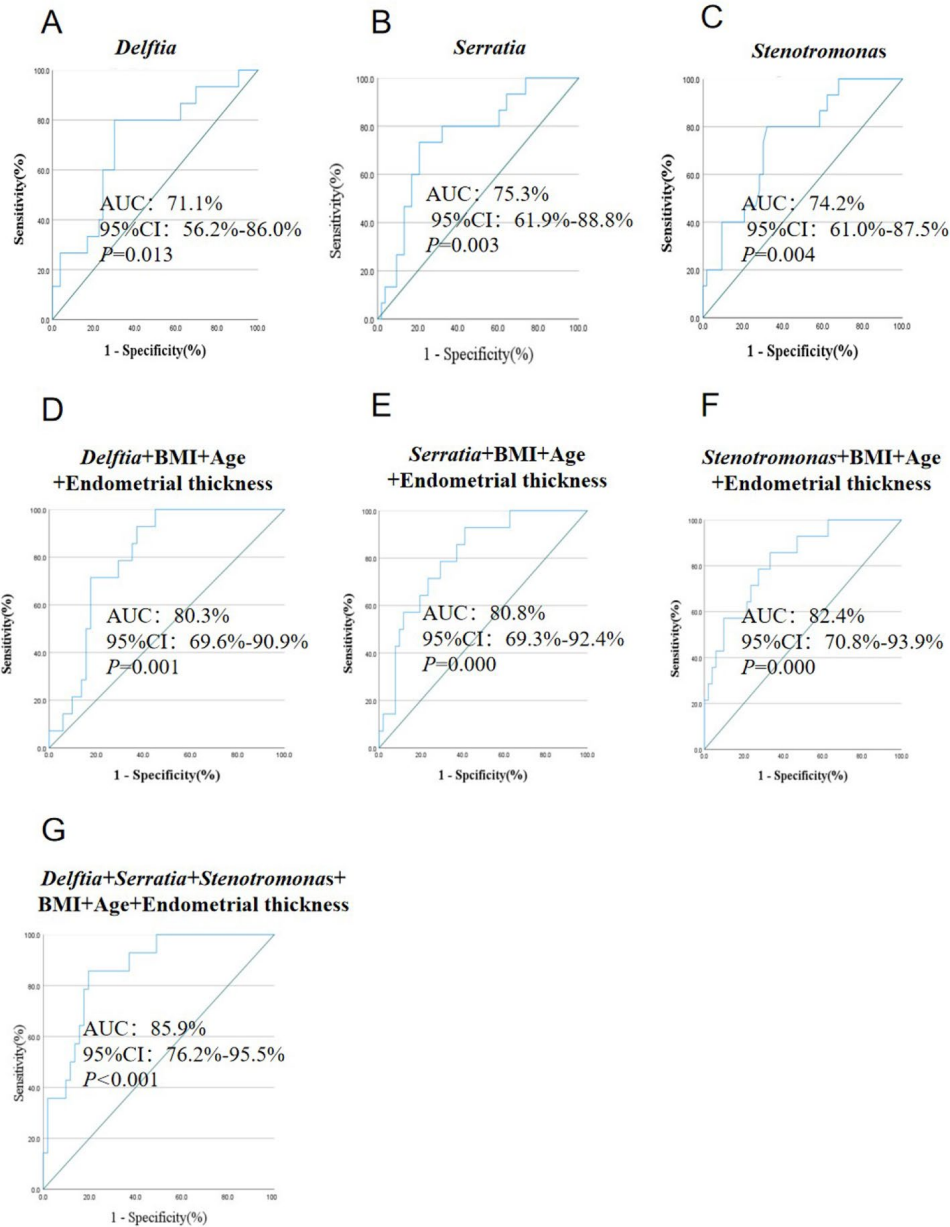
ROC curve for *Delftia*, *Serratia* and *Stenotrophomonas* abundance for predicting endometrial hyperplasia

To evaluate the potential diagnostic value of *Delftia*, *Serratia* and *Stenotrophomonas*, ROC curves were generated to differentiate between EH and normal endometrial samples (Fig. 4). The AUC for the *Delftia* group was 71.1%, with a 95% confidence interval (CI) ranging from 56.2% to 86.0% (Fig. 4A). The AUC for the *Serratia* group was 75.3%, with a 95% confidence interval (CI) ranging from 61.9% to 88.8% (Fig. 4B). Similarly, the AUC for the *Stenotrophomonas* group was 74.2%, with a 95% confidence interval (CI) ranging from 61.0% to 87.5% (Fig. 4C). The AUC was improved if the clinical features, such as the age and BMI of the patients, the thickness of endometrium factored in (Fig. 4D-G). These findings suggest that microbiome-associated biomarkers, such as *Delftia*, *Serratia* and *Stenotrophomonas*, may represent a promising alternative approach for distinguishing between EH and normal endometrial samples.

## Discussion

The microbiome of the female genital tract exerts a pivotal influence on women's health, exerting profound effects on both benign conditions and malignancies. Benign conditions encompass a spectrum ranging from endometriosis and bacterial vaginosis to infertility, chronic pelvic pain, preterm birth, and miscarriage. Meanwhile, cancers such as ovarian, cervical, and endometrial cancers are also significantly influenced by the composition and dynamics of the female genital tract microbiome [6, 12, 13]. The immune system is integral in moderating the microbiome's impact on women's health and endometrial stability by recognizing and interacting with microbiota to uphold microbial balance. Immune cells, including NK cells, macrophages, and dendritic cells, play a pivotal role in regulating immune responses to distinguish between beneficial and harmful microbes. This immune-microbiome interplay is crucial for averting reproductive complications, preserving fertility, and ensuring overall well-being, with dysregulation potentially leading to conditions like infertility, miscarriage, and obstetric issues [14, 15].

This study revealed that the most dominant taxa in the uterus were Proteobacteria, Firmicutes, and Bacteroidetes. Despite the limited information available on the microbiota of upper female reproductive tract tissues, our findings align with previous studies which have reported Proteobacteria and Bacteroidetes were the top two taxa in the uterus of European descent [16]. Our research has identified that Achromobacter, Brevundimonas, *Delftia*, Pandoraea, and Sphingomonas are the predominant genera of microorganisms found in the uterine cavity of women with or without EH. Various studies have also demonstrated that *Delftia* was among the most prevalent genera of microorganisms in the endometrial microbiome [17–19]. Furthermore, the absence of *Delftia* in the vaginal region distinguishes it as a distinct microbe found exclusively in the upper reproductive tract tissues of females [18, 20].

Our study revealed an intriguing finding that patients with EH had an elevated abundance of Proteobacteria, specifically *Delftia*, in the uterus compared to those with a benign endometrium. *Delftia* was found to be the predominant taxon present in the uterine microbiota of patients with endometrial hyperplasia (EH), whereas it was not the most abundant taxon observed in the uterine microbiota of patients without EH. In benign endometrium, the mean relative abundance of *Delftia* was 0.114 (*N* = 53), whereas in EH it was 0.170 (*N* = 15). Our finding suggested a potential association between the presence of *Delftia* and the development of EH. Previous studies demonstrated different results that infertile women had a significantly higher or lower abundance of *Delftia* compared to fertile women [17, 21]. The abundance of *Delftia* was found to be higher in the endometrium of patients diagnosed with endometriosis [22]. The composition of the uterine microbiome in individuals with endometrial cancer was found to exhibit varying relative abundances of *Delftia*, depending on factors such as obesity and race, in both women and mice [23]. The aforementioned discoveries suggest the possibility of a correlation between *Delftia* and aspects of reproductive well-being.

Patients with EH were found to had an elevated abundance of *Serratia* in the uterus compared to those without EH in our study. *Serratia* was found to be related with maternal or infant Infections [24–26]. We discovered new information about *Serratia*.

In our study, patients with EH had an elevated abundance of *Stenotrophomonas* in the uterus compared to those without EH. This finding is consistent with a previous study that reported a higher relative abundance of *Stenotrophomonas* in women with endometrial cancer/endometrial hyperplasia [27].

The most import finding of our study was that the higher abundance of *Delftia*, *Serratia* and *Stenotrophomonas* bacteria in the endometrium may be indicative of endometrial hyperplasia. Our finding shed light on potential biomarkers for EH and contribute to advancements in the field of uterine microbiome research.

In our study, several limitations merit discussion. Factors such as sexual habits, hygiene practices, and BMI are known to influence the uterine microbiota. However, quantifying sexual habits or hygiene practices poses challenges. Additionally, our data revealed a trend towards higher BMI in the control group, although this trend did not reach statistical significance. This finding contrasts with the conventional belief that higher BMI is a risk factor for endometrial pathologies [28]. One limitation of our study was the relatively small sample size, consisting of 53 patients with benign endometrium and 15 patients with EH. While our findings suggest potential trends that warrant further investigation, it is important to note that our results may not be fully representative of the broader population. Therefore, it is necessary to conduct future studies with larger cohorts of patients in order to confirm and expand upon our findings. In our study, we opted for 16S rRNA sequencing over Shotgun Metagenomic Sequencing due to its targeted approach for characterizing specific microbial taxa. While Shotgun Metagenomic Sequencing offers a broader view of microbial communities, it is more susceptible to biases during library preparation and data analysis [29]. Further studies utilizing both techniques in parallel may provide a more robust understanding of the female genital tract microbiome and its implications for women's health. Our study is also limited by the potential contamination of endocervical mucus in Pipelle biopsy samples, resulting in a mixed microbial composition from both endometrial and endocervical sources. While efforts were made to minimize contamination during sample collection and processing, the presence of endocervical mucus introduces variability in microbial composition analysis. This consideration is crucial when interpreting the results, as it may affect the accuracy and specificity of findings related to the endometrial microbiota. Future research could explore alternative sampling techniques to address this limitation and achieve a more precise characterization of the endometrial microbiome.

## Conclusions

In conclusion, the evidence in our study suggested a unique microbiome pattern in individuals with endometrial hyperplasia, a condition that involves the higher abundance of *Delftia* and *Stenotrophomonas* in the endometrial microbiome. Our research indicates that the presence of high levels of *Delftia* and *Stenotrophomonas* in the uterine cavity is associated with the development of endometrial hyperplasia. These findings have significant implications for the identification of microbiota biomarkers that could aid in the early detection of endometrial cancer or endometrial hyperplasia.

## Methods

### Study population

This study was conducted at the Women’s Hospital, School of Medicine, Zhejiang University, from April 2021 to March 2022. The inclusion criteria for participants were as follows: (1) individuals aged between 25 and 60 years, (2) women undergoing endometrial biopsy due to abnormal vaginal bleeding or abnormal ultrasound findings, and (3) pathology-confirmed benign endometrium or endometrial hyperplasia (EH). Patients who met any of the following criteria were excluded from the study: (1) pregnant or nursing women, (2) patients who used antibiotics or micro-ecologies within the past three months, (3) history of cancer, and (4) pathology-confirmed endometrial carcinoma.

The procedure for acquiring endometrial specimens was conducted in an outpatient clinic and comprised three primary stages. Initially, standard disinfection of the external genitalia, vagina, and cervix was performed to mitigate the influence of vaginal microbiota. Sterile cotton-headed swabs with polypropylene backing were utilized to disinfect the uterine canal three times, aiming to minimize the influence of microbiota from the cervical canal. Subsequently, a Pipelle device (e.g., Pipelle, Cooper Surgical, Inc., Trumbull, CT, USA) was employed to procure endometrial tissue or mucus for microbial analysis. The harvested endometrial tissue was stored at -80°C until DNA extraction was conducted. Finally, endometrial sampling was performed via curettage to obtain samples suitable for pathological examination. The collected endometrial tissues were fixed in 10% buffered formalin and transported to the pathology laboratory for histopathological studies. The procedures were conducted by a senior obstetrics and gynecology (OBGYN) specialist with over 10 years of experience. No instances of surgical complications occurred in any patients, such as uterine perforation, inability to access the uterine cavity, or failure to procure sufficient endometrial tissue for histological examination. The obtained specimens were evaluated by two independent experts in gynecologic histopathology following guidelines for the classification of entities related to endometrial pathological findings.

This study was approved by the ethics review board of the Human Ethics Committee of the Women’s Hospital, School of Medicine, Zhejiang University. After the study procedures associated with the participation in this study were explained, written informed consent was obtained from all participants before data acquisition. This study was conducted in accordance with the principles of the Declaration of Helsinki.

### DNA extraction and 16S rRNA gene sequencing for bacterial communities

Total genomic DNA was extracted from samples using the SDS method. The concentration and purity of the DNA were assessed using 1% agarose gels. Subsequently, the DNA was diluted to a concentration of 1 ng/μl using sterile water. To detect any possible contaminants from reagents or other sources, a blank buffer control was included in each extraction. All negative controls showed no detectable DNA and failed the library preparation, hence were excluded from sequencing.

The V3 and V4 regions of the 16S rRNA gene were amplified using universal primers 341F (5'-CCTAYGGGRBGCASCAG–3') and 806R (5'GGACTA-CNNGGGTATCTAAT-3') 20. The amplification was carried out to target these regions for analysis.

### 16S rRNA gene compositional analysis

The raw paired-end sequences from EH and benign endometrial tissue samples were imported into the QIIME platform (version 2019.4, available at https://qiime2.org). These sequences were demultiplexed and then denoised using the DADA2 algorithm, which enabled the identification of representative amplicon sequencing variants (ASVs). These ASVs were used to generate a fragment-insertion phylogenetic tree.

To determine the taxonomic classification of the ASVs, they were submitted to a pretrained naive Bayes classifier that had been trained on the full-length 99% Greengenes 13_8 reference. This classification process allowed for accurate identification of the microbial species present in the samples.

### Statistical analysis

In this study, the diversity and composition of endometrial microbiota in two groups were analyzed using QIIME2 (version 2019.4). Before diversity analyses, feature tables were evenly rarefied with 30,000 sampling depths by random subsampling. Alpha diversity was measured using Shannon's diversity index and Simpson's diversity index. Beta diversity was evaluated using weighted UniFrac distance matrices calculated with QIIME2, which were represented as principal coordinates (PCoA) to examine bacterial community composition. The permutational multivariate analysis of variance (PERMANOVA) and permutational analysis of multivariate dispersions (PERMDISP) tests were conducted to evaluate whether the within-group distances were different from the between-group distances.

The significant differences in microbial taxon abundances were analyzed using the ANCOM-II23 method between the two groups, with a cutoff of 0.6 to identify differentially abundant operational taxonomic units (OTUs) or taxa. The final significance was expressed in an empirical distribution of W. Fundamental statistical analyses were performed using SPSS software (version 21, IBM). For quantitative variables, means and standard deviations were calculated when the distribution was normal, and medians and interquartile ranges were calculated when the distribution was non-normal. The group differences were analyzed using Student's t-test or Mann–Whitney U test for quantitative data. Categorical variables were expressed as numbers (%) and analyzed using the chi-squared test. A two-sided *p*-value < 0.05 was considered statistically significant.

Receiver operating characteristic curves (ROC) were created to assess the diagnostic value of abundant microbes in EH. Identification markers with an area under the ROC curve (AUC) greater than 0.7 were considered successful.

## Data Availability

The datasets generated and analysed during the current study are available via NCBI under the project number PRJNA1004537 or https://www.ncbi.nlm.nih.gov/sra/PRJNA1004537.

## References

[1] Singh S, Pavuluri S, Lakshmi BJ, Biswa BB, Venkatachalam B, Tripura C (2020). Molecular characterization of Wdr13 knockout female mice uteri: a model for human endometrial hyperplasia. Sci Rep.

[2] Liang Y, Lin B, Ye Z, Chen S, Yu H, Chen C (2020). Triple-high expression of phosphatase and tensin homolog (PTEN), estrogen receptor (ER) and progesterone receptor (PR) may predict favorable prognosis for patients with Type I endometrial carcinoma. J Cancer.

[3] Emons G, Beckmann MW, Schmidt D, Mallmann P (2015). New WHO Classification of endometrial hyperplasias. Geburtshilfe Frauenheilkd.

[4] Baker JM, Chase DM, Herbst-Kralovetz MM (2018). Uterine microbiota: residents, tourists, or invaders?. Front Immunol.

[5] Kyono K, Hashimoto T, Kikuchi S, Nagai Y, Sakuraba Y (2018). A pilot study and case reports on endometrial microbiota and pregnancy outcome: an analysis using 16S rRNA gene sequencing among IVF patients, and trial therapeutic intervention for dysbiotic endometrium. Reprod Med Biol.

[6] Łaniewski P, Ilhan ZE, Herbst-Kralovetz MM (2020). The microbiome and gynaecological cancer development, prevention and therapy. Nat Rev Urol.

[7] Khan I, Nam M, Kwon M, Seo S, Jung S, Han JS (2019). LC/MS-based polar metabolite profiling identified unique biomarker signatures for cervical cancer and cervical intraepithelial neoplasia using global and targeted metabolomics. Cancers.

[8] van den Helder R, Steenbergen RDM, van Splunter AP, Mom CH, Tjiong MY, Martin I (2022). HPV and DNA methylation testing in urine for cervical intraepithelial neoplasia and cervical cancer detection. Clin Cancer Res Off J Am Assoc Cancer Res.

[9] Pichon M, Pichard B, Barrioz T, Plouzeau C, Croquet V, Fotsing G (2020). Diagnostic accuracy of a noninvasive test for detection of helicobacter pylori and resistance to clarithromycin in stool by the Amplidiag H. pylori+ClariR real-time PCR assay. J Clin Microbiol.

[10] Zhao P, Ma Z, Jiao Y, Yan Y, Li S, Du X (2022). Laparoscopic and endoscopic cooperative surgery for early gastric cancer: Perspective for actual practice. Front Oncol.

[11] Walther-António MRS, Chen J, Multinu F, Hokenstad A, Distad TJ, Cheek EH (2016). Potential contribution of the uterine microbiome in the development of endometrial cancer. Genome Med.

[12] Salliss ME, Farland LV, Mahnert ND, Herbst-Kralovetz MM (2021). The role of gut and genital microbiota and the estrobolome in endometriosis, infertility and chronic pelvic pain. Hum Reprod Update.

[13] Salmeri N, Sinagra E, Dolci C, Buzzaccarini G, Sozzi G, Sutera M (2023). Microbiota in irritable bowel syndrome and endometriosis: birds of a feather flock together—a review. Microorganisms.

[14] Agostinis C, Mangogna A, Bossi F, Ricci G, Kishore U, Bulla R (2019). Uterine immunity and microbiota: a shifting paradigm. Front Immunol.

[15] Salmeri N, Gennarelli G, Vanni VS, Ferrari S, Ruffa A, Rovere-Querini P (2023). Concomitant autoimmunity in endometriosis impairs endometrium-embryo crosstalk at the implantation site: a multicenter case-control study. J Clin Med.

[16] Verstraelen H, Vilchez-Vargas R, Desimpel F, Jauregui R, Vankeirsbilck N, Weyers S (2016). Characterisation of the human uterine microbiome in non-pregnant women through deep sequencing of the V1–2 region of the 16S rRNA gene. PeerJ.

[17] Barinova VV, Kuznetsova NB, Bushtyreva IO, Oksenyuk OS, Shatalov AE (2021). Uterine microbiome in healthy fertile women and in case of endometrium pathology in multiple failures of in-vitro-fertilization programs. Period Tche Quimica.

[18] Riganelli L, Iebba V, Piccioni M, Illuminati I, Bonfiglio G, Neroni B (2020). Structural variations of vaginal and endometrial microbiota: hints on female infertility. Front Cell Infect Microbiol..

[19] Sobstyl M, Brecht P, Sobstyl A, Mertowska P, Grywalska E (2022). The role of microbiota in the immunopathogenesis of endometrial cancer. Int J Mol Sci.

[20] Hawkins GM, Clark LH, Sullivan SA, Keku TO, Brewster WR, Bae-Jump VL (2019). Impact of ethnicity and obesity on the uterine microbiome in women and mice with endometrial cancer. Gynecol Oncol.

[21] Ichiyama T, Kuroda K, Nagai Y, Urushiyama D, Ohno M, Yamaguchi T (2021). Analysis of vaginal and endometrial microbiota communities in infertile women with a history of repeated implantation failure. Reprod Med Biol.

[22] Wei W, Zhang X, Tang H, Zeng L, Wu R (2020). Microbiota composition and distribution along the female reproductive tract of women with endometriosis. Ann Clin Microbiol Antimicrob.

[23] Clark LH, Keku TO, McCoy NA, Hawkins G, Bae-Jump VL, Brewster WR (2017). Alterations in the uterine microbiome in patients with early endometrial cancer: variations by ethnicity and obesity. J Clin Oncol..

[24] Das AM, Paranjape VL, Pitt TL (1988). Serratia marcescens infection associated with early abortion in cows and buffaloes. Epidemiol Infect.

[25] Erenberg M, Yagel Y, Press F, Weintraub AY (2017). Chorioamnionitis caused by Serratia marcescens in a healthy pregnant woman with preterm premature rupture of membranes: A rare case report and review of the literature. Eur J Obstet Gynecol Reprod Biol..

[26] van Ogtrop ML, van Zoeren-Grobben D, Verbakel-Salomons EM, van Boven CP (1997). Serratia marcescens infections in neonatal departments: description of an outbreak and review of the literature. J Hosp Infect.

[27] Chao A, Chao A-S, Lin C-Y, Weng CH, Wu R-C, Yeh Y-M (2022). Analysis of endometrial lavage microbiota reveals an increased relative abundance of the plastic-degrading bacteria Bacillus pseudofirmus and Stenotrophomonas rhizophila in women with endometrial cancer/endometrial hyperplasia. Front Cell Infect Microbiol.

[28] Giannella L, Grelloni C, Bernardi M, Cicoli C, Lavezzo F, Sartini G (2024). Atypical endometrial hyperplasia and concurrent cancer: a comprehensive overview on a challenging clinical condition. Cancers.

[29] Durazzi F (2021). Comparison between 16S rRNA and shotgun sequencing data for the taxonomic characterization of the gut microbiota. Sci Rep..

